# Computational studies deciphered the role of key genes and associated networks regulating the defense mechanism in chickpea under *Fusarium oxysporum* f. sp. ***ciceris*** induced wilt condition

**DOI:** 10.1080/15592324.2026.2631915

**Published:** 2026-02-18

**Authors:** Anjali Chaudhary, Annamalai Arunachalam, P. T. V. Lakshmi

**Affiliations:** a Phytomatics Lab, Department of Bioinformatics, Pondicherry University, Puducherry, India; b Department of Food Science and Technology, Pondicherry University, Puducherry, India

**Keywords:** CAZymes, GH28, Fusarium wilt, R-genes, hub genes, transcription factors

## Abstract

*Cicer arietinum*, a nutritionally rich legume and a key Rabi crop, is severely impacted by vascular wilt caused by *Fusarium oxysporum* f. sp. *ciceris*, leading to substantial yield loss. To understand molecular reprogramming, a transcriptomic dataset of chickpea seedling roots exposed to fungal infection at 7 and 12 d post-inoculation (dpi) was analyzed. The comparison was made between the control and stressed at 7 and 12 dpi (C_7 vs S_7 and C_12 vs S_12) to be compared against stressed at 7 vs 12 dpi (S_7 vs S_12). The analyses included differentially expressed gene (DEGs) patterns, protein interaction networks, hub genes, carbohydrate-active enzymes (CAZymes), resistance genes (R-genes), and transcription factor (TF) identifications at two different time points. It revealed a total of 894, 867, and 535 significant DEGs respectively, which were associated mainly with cell-wall modification, membrane components, pectinesterase inhibitor activity, terpene synthase activity, and the biosynthesis of secondary metabolites. Further, the protein‒protein interaction network revealed that hub genes associated with chloroplast-localized reactive oxygen species scavenging activity and CAZyme, particularly glycosyl hydrolase 28 (GH28), to be involved during the early phase of infection. R-genes belonging to the classes of KIN (kinase domain), RLP (receptor-like protein), and RLK (receptor-like kinase) were significantly expressed while TFs, bHLH (basic helix–loop–helix), and GeBP (glabrous-enhancer-binding protein) were downregulated with prolonged infection. Hence, the overall study identified the key regulators, orchestrating the defense molecular mechanisms in relation to the time course of infection by the pathogen.

## Graphical abstract

**Figure:**
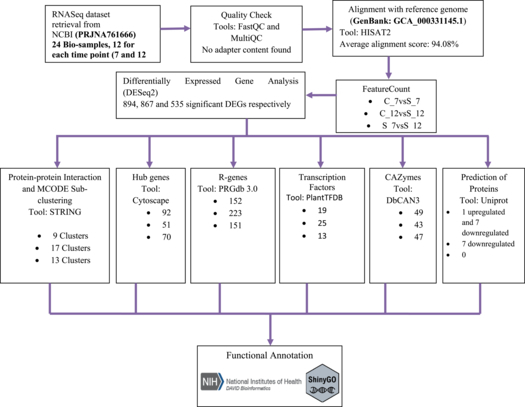

## Introduction

Chickpea (*Cicer arietinum* L.) an important low-calorie food legume that is valued for its high protein, dietary fiber, and complex carbohydrates, significantly contributes to global consumer demand, with India being the topmost producer, and is expected to export $ 19.19 billion by 2027, contributing to a 6.5% share of the global market (https://www.icar-iipr.org.in/chickpea-crop/;^1^). Perhaps, in India, the states of Maharashtra (25.97%), Madhya Pradesh (18.59%), Rajasthan (20.65%), and Gujarat (10.10%) contribute the largest production of both *desi* and *Kabuli* types, which differ in their physical and genetic traits. Owing to its high nutritional value, chickpea has gained attention as one of the important functional foods, and its consumption is reported to increase the health benefits to humankind, making its yield stability a critical priority, as envisioned for 2050 (https://www.icar-iipr.org.in/chickpea-crop/). However, since chickpea productivity is adversely affected by both abiotic and biotic stresses, fungi, as one of the obstructive organisms, pose a major threat because 67 out of 172 fungi are reported to affect chickpea,^2^ causing severe diseases, including wilt caused by *Fusarium oxysporum* f. sp. *ciceris.*^3^ Hence, understanding the pathogenesis of Fusarium wilt (FW) is considered the need of the hour. FW, a vascular disease caused by *F. oxysporum* f. sp. *ciceris* (Foc), is a soil-borne, necrotrophic fungus ranked among the world's top five most destructive pathogens due to its scientific and economic significance.^4^ It exists in two pathotypes (yellowing and wilt-causing) and comprises eight pathogenic races (0, 1, 1B/C, 2, 3, 4, 5, and 6), of which Race 1 is the most prevalent in India and is a major concern for disease management.^5^ The fungus infects through the roots of the host plant, colonizes the xylem vessels, and utilizes the plant's nutrients for rapid mycelial growth. This extensive colonization clogs the transport system, obstructing water, and nutrients from reaching the aerial parts of the plant, ultimately leading to its death.^6^ Foc reproduces asexually through microconidia, macroconidia, and long-surviving chlamydospores that can persist in soil for years in the absence of a host plant.^7^^,^^8^ This long-term soil survival, integrated with pathogenic variability and mutability, poses a primary challenge for plant breeders, as it can lead to the breakdown of natural resistance over the course of progressive Foc infection.

However, controlling the disease using conventional methods becomes ineffective.^3^^,^^9^ Therefore, employing wilt-resistant cultivars would be the most efficient and sustainable method for managing the disease. In view of this, various “omics” approaches have been applied to understand the molecular mechanisms underlying the chickpea‒Foc pathosystem. To develop chickpea cultivars resistant to Foc, several molecular and genetic approaches have been employed, including marker-assisted gene mapping,^4^ quantitative trait loci (QTL),^10^ candidate gene identification and differential gene expression. Multi-omics strategies such as metabolomics and transcriptomics have been widely utilized to unravel the stress–response mechanisms in chickpea.^8^ Proteomic techniques such as 2D PAGE, MALDI-TOF MS, and MS/MS have also facilitated the identification of proteins associated with defense signaling,^5^ while the LongSAGE study provided insights into novel genes involved in lignification, plant defense, and R-gene-mediated resistance.^9^ Transcription factor-associated pathways have further contributed insights into chickpea–Foc interactions.^11^ Despite these advances, the molecular basis of chickpea resistance to Foc remains largely unexplored, creating a lacuna in elucidating the regulatory mechanisms that confer disease resistance. Therefore, current biological and chemical disease management approaches have been largely ineffective, highlighting the need to utilize R-genes and CAZymes that interact with fungal effector proteins to activate effector-triggered immunity (ETI), as has been demonstrated in tomato.^12^^,^^13^

Although these studies indicate their role in understanding the molecular mechanisms to an extent seems insufficient to decipher the molecular interactions involved in disease resistance and perhaps to develop/improve FW-resistant cultivars in chickpea, information on the CAZymes, R-genes, transcription factors, protein‒protein interaction networks, and hub genes is essential to improve the understanding of the regulatory mechanisms underlying chickpea defense responses. Therefore, the present study was hypothesized to address these knowledge gaps by analyzing a publicly available RNA-Seq dataset generated from *F. oxysporum* f. sp*. ciceris* infected chickpea roots at 7 and 12 dpi. The dataset retrieved from the NCBI SRA were examined to identify differentially expressed genes (DEGs) and to predict CAZymes, R-genes, transcription factors, protein‒protein interactions, and hub genes associated with Fusarium wilt resistance and disease progression. This study aims to advance understanding of the molecular events shaping chickpea–Foc interactions at distinct infection stages.

## Methodology

### Data collection

The transcriptome dataset from chickpea roots comprised 24 biosamples and was retrieved from the NCBI SRA database (Bioproject ID: PRJNA761666). These dataset generated from the roots of chickpea seedlings exposed to two different time points of seventh and twelfth day post inoculation (dpi) of fungus *F. oxysporum* f. sp. *ciceris,*^11^ were selected for comparing their profile with that of the control separately and, between them as, control (C) vs stressed (S) at seventh dpi (C_7 vs S_7); control vs stressed at twelfth dpi (C_12 vs S_12) and stressed at seventh vs at twelfth dpi (S_7 vs S_12) (Supplementary File, S1-Table 1).

### Preprocessing, quality check of RNA-Seq data and preparation

RNA-Seq reads were quality checked using FastQC, and then the HISAT2 index for the *C. arietinum* reference genome (GenBank: GCA_000331145.1) was constructed using “hisat2-build,” which uses a graph-based alignment approach (graph Ferragina Manzini index), followed by alignment of paired-end RNA-Seq reads to the indexed genome using HISAT2 with strand-specific and downstream analysis parameters generating output files in sequence alignment map (SAM) format.^14^ MultiQC was subsequently performed, and the data were sorted into binary alignment matrix (BAM) format using Samtools. Finally, transcript raw read counts were extracted using FeatureCounts from the Subread package to generate the count matrix by considering the strand-specific nature of the RNA-Seq library (-s1 for forward and -s2 for reverse-stranded libraries), ensuring proper mapping of reads to their transcribed genes^15^ (Supplementary File, S1-Table 2).

### Differential gene expression, gene ontology (GO), and KEGG pathway analyses

DESeq2 (version 1.42.1) of the R package, with the cut-off value of log_2_ fold change of ≥|1.5| and *p*-value < 0.05, was used to identify significant DEGs. Furthermore, functional annotation was performed using NCBI DAVID, which employs an agglomerative clustering algorithm.^16^^,^^17^ The gene ontology categories biological process (BP), molecular function (MF), cellular component (CC), and KEGG pathways were visualized using ggplot2 (v3.5.1). Transcription factors and associated pathways were identified via local BLASTx against the Plant Transcription Factor Database (PlantTFdb)^18^ with parameters, percentage identity ≥ 80, e-value ≤ 0 and bit score > 200.^19^ Additionally, R-gene classes and domains were determined through the DRAGO pipeline in the Plant Resistance Gene database (PRGdb 3.0).^20^ Moreover, carbohydrate-active enzyme (CAZyme) genes were classified by aligning DEG sequences against dbCAN3^21^ using the default parameters.

### Protein–protein interaction network construction

Protein–protein interaction network of DEGs was constructed using STRING (version 12.0), with a confidence score > 0.4. Then, networks were visualized using Cytoscape (version 3.2.1)^22^ and analyzed for topological properties using Network Analyzer. Further, sub-clustering was performed using MCODE (molecular complex detection) clustering plugin with default parameters: degree cutoff = 2, k-core = 2, and maximum depth = 100. Moreover, common hub genes identified were extracted for top 100 genes based on degree of connectivity, maximum neighborhood component (MNC), maximal clique centrality (MNC), and edge percolated component (EPC) using the Cytohubba plugin of Cytospace, and finally, the common hub genes across C_7 vs S_7, C_12 vs S_12, and S_7 vs S_12 were subjected to functional enrichment by ShinyGO.^23^ Additionally, DEGs were also mapped against the UniProt database to identify their roles in host‒pathogen interactions.^24^

## Results

### Preprocessing and analysis of differentially expressed genes

**Figure 1.**
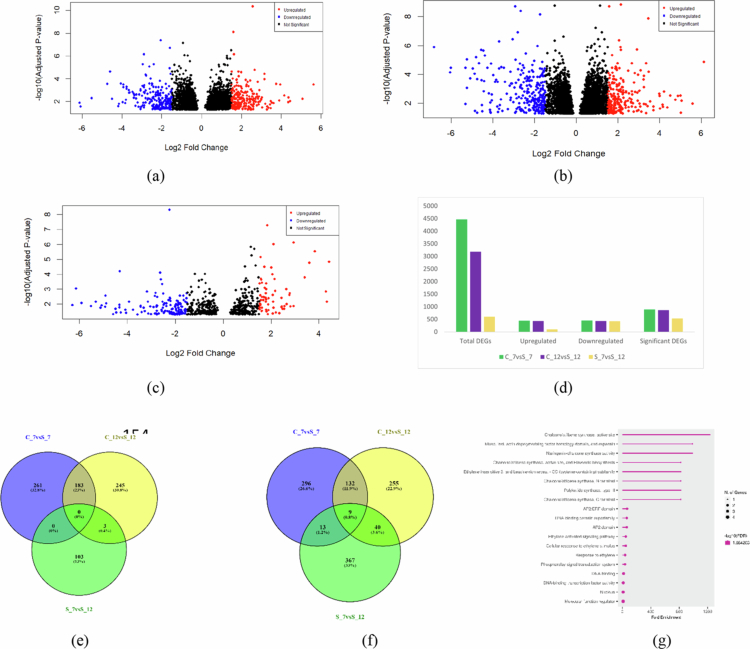
(a) Differentially expressed genes. Volcano plot represents the expression of differential genes compared at different time points; (b) C_7 vs S_7; (c) C_12 vs S_12; (d) S_7 vs S_12; (e) overall comparison of differentially expressed genes within the time points, upregulated; (f) downregulated; and (g) functional enrichment of common downregulated DEGs between C_7 vs S_7, C_12 vs S_12, and S_7 vs S_12. The red color in the volcano plot represents upregulated DEGs, whereas the blue color indicates downregulated expressed genes, and black dots occurring as a clumsy cluster display insignificantly differentially expressed genes. C_7 vs S_7: control vs stressed (Foc-infected) conditions at 7 d post inoculation; C_12 v sS_12: control vs stressed conditions at 12 d post inoculation; and S_7 vs S_12: harvest on 7 d stressed compared with 12 d stressed; and Foc: *Fusarium oxysporum* f. sp. *ciceris*.

The quality check was satisfactory across all samples, with no adapter contamination and with an average alignment score of 94.08% when mapped with the chickpea reference genome (Supplementary File, S1). At 7 dpi (C_7 vs S_7), a total of 894 genes were significantly differentially expressed, comprising 444 upregulated and 450 downregulated, whereas on day 12 (C_12 vs S_12), 867 DEGs were significantly expressed, where 431 were upregulated and 436 were downregulated. However, 535 DEGs with 106 upregulated and 429 downregulated were significantly expressed when stress at different time points (S_7 vs S_12) was compared (Figure 1d).

Since common genes may provide an insight into the resistance mechanism to understand defense responses and recognize potential targets for enhancing resistance against fungal infection,^25^ common DEGs between C_7 vs S_7 and C_12 vs S_12 were analyzed, which revealed 183 upregulated and 132 downregulated genes comprising 26.4% and 11.9%, respectively. However, cross-examination between the stressed during 7–12 d (S_7 vs S_12) against the controls (C_7 vs S_7 and C_12 vs S_12) indicated a high number of genes were downregulated with longer duration of stress, whereas only nine downregulated (0.8%) DEGs were identified as common under all three conditions (Figure 1e and f; Supplementary File, S2). Further, the volcano plot also displayed the expression matrix of the genes, with red showing the significantly differentially expressed genes, i.e., upregulated genes (log2-fold change ≥ 1.5 and *p*-value below 0.05), and downregulated genes (log2-fold change ≥ −1.5 and *p*-value below 0.05), whereas black color corresponds to genes with no statistically significant differential expression (Figure 1a–c).

### GO function and KEGG pathway enrichment analysis

**Figure 2.**
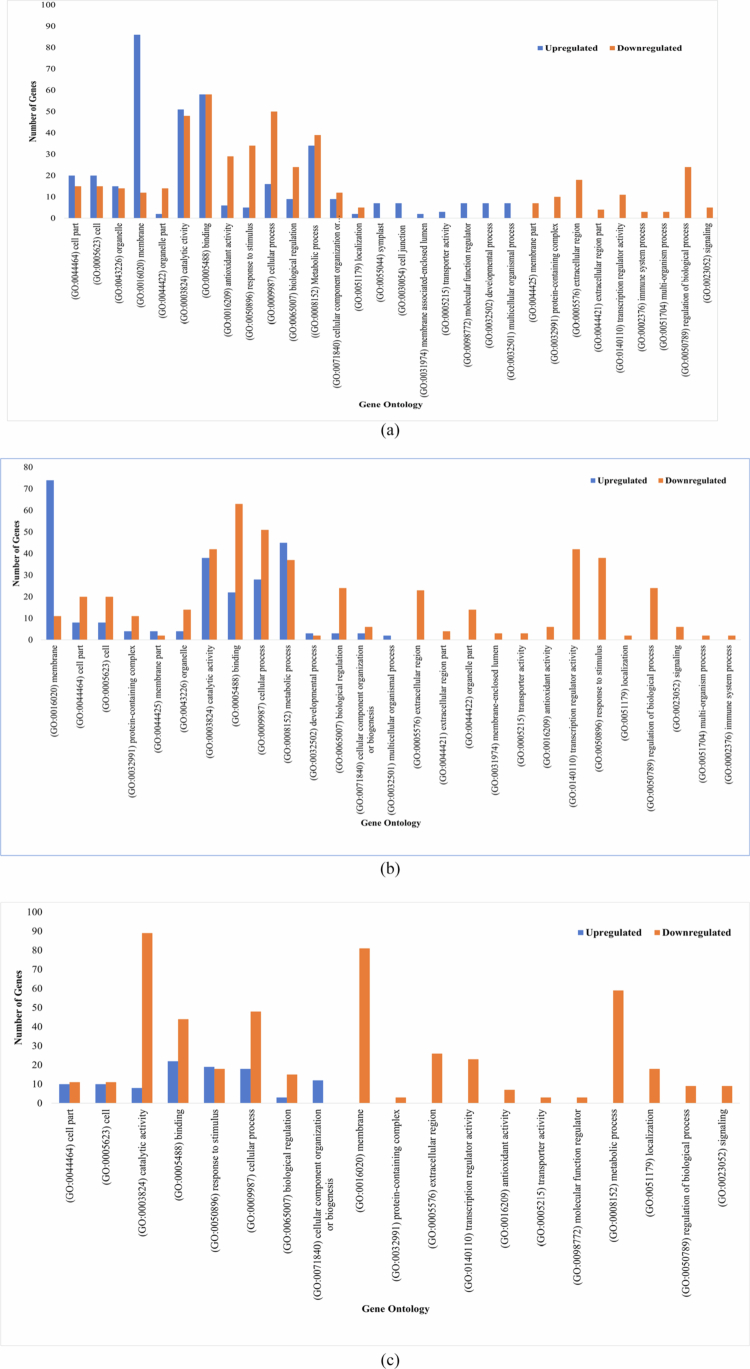
Comparison of the gene ontology for the differentially expressed genes; (a) C_7 vs S_7 (control vs stressed (Foc-infected) conditions on 7 d post inoculation); (b) C_12 vs S_12 (control vs stressed conditions on 12 d post inoculation); and (c) S_7 vs S_12 (harvest on 7 d stressed compared with 12 d stressed).

**Figure 3.**
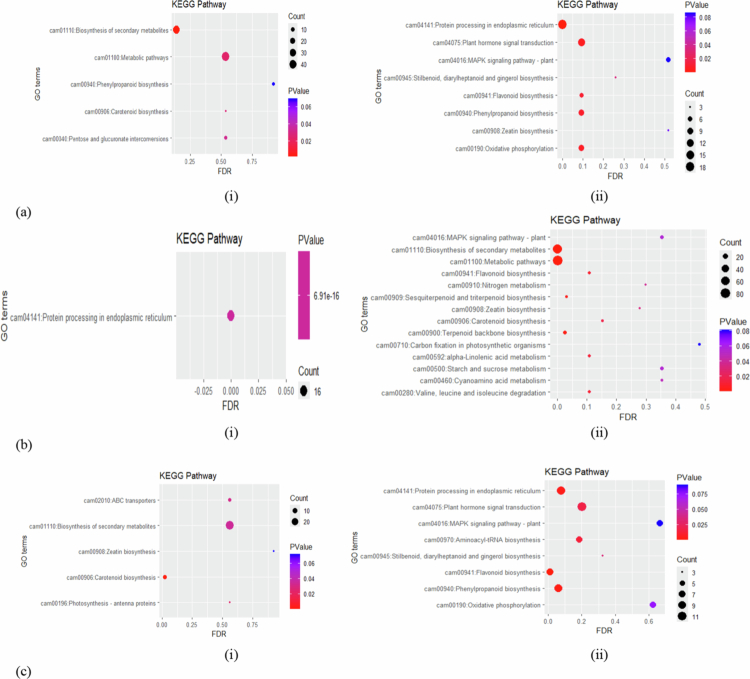
Comparison of the pathways analyzed through KEGG for differentially expressed genes (a) C_7 vs S_7, (i) upregulated DEGs (ii) downregulated DEGs; (b) C_12 vs S_12, (i) upregulated DEGs (ii) downregulated DEGs; and (c) S_7 vs S_12, (i) upregulated DEGs (ii) downregulated DEGs. C_7 vs S_7: control vs stressed (Foc-infected) conditions on 7 d post inoculation; C_12 vs S_12: control vs stressed conditions on 12 d post inoculation and S_7 vs S_12: harvest on 7 d stress compared with 12 d stressed.

GO enrichment analysis revealed distinct functional patterns across stress conditions. Under C_7 vs S_7, a greater number of upregulated DEGs were enriched across BP (223), CC (275), and MF (219), with 84 genes associated with KEGG pathways. In contrast, fewer downregulated DEGs were represented within the same categories, comprising 202 (BP), 217 (CC), 192 (MF), and 87 (KEGG) genes, respectively (Figures 2a and 3a). However, at the later point C_12 vs S_12, enrichment patterns were more balanced between upregulated (729) and downregulated (689) genes, of which approximately 200 upregulated and 186 downregulated DEGs were associated with BP, while 193 and 197 corresponded to CC formation and disruption, while molecular processes enrichment involved 254 upregulated and 218 downregulated DEGs, with KEGG pathway analysis identifying 82 and 88 genes, respectively (Figures 2b and 3b). Furthermore, the condition S_7 vs S_12 showed pronounced transcriptional repression, with 917 downregulated DEGs compared to only 181 upregulated genes. Functional annotation revealed that upregulated DEGs were modestly enriched across BP (56), CC (41), MF (57), and KEGG pathways (27), whereas downregulated DEGs were strongly enriched in BP (236), CC (234), and MF (321) pathways and KEGG pathways (126), highlighting the widespread suppression of functional pathways under prolonged stress (Figures 2c and 3c).

Interestingly, at 7 dpi, a significant shift in gene expression was observed, indicating strong regulation of metabolic and cellular activities, which potentially influence cell component biogenesis in response to infection. A total of 113 DEGs were identified, of which 50 regulated “cellular process” (GO:0009987), 39 corresponded to “metabolic process” (GO:0008152), and 24 genes decoded for “biological regulations” (GO:0065007) significantly downregulating the overall process. Additionally, gene associated with “cellular component biogenesis” (9 DEGs, GO:0071840) and “stimulation” (34 DEGs, GO:0050896) were predominantly downregulated. However, in addition to the decline, a small subset of genes (7 DEGs, GO:0032502) supported seedling development and were considered key players in overcoming infection. Furthermore, several defense-related genes (GO:0023052) were also downregulated, indicating reduced immune-associated signaling. Moreover, to combat infection, certain cellular genes were over-expressed, providing the first line of defense, which included 86 DEGs corresponding to “membrane” growth and activities (GO:0016020) along with 35 DEGs (GO:0005623) associated with the formation of the cell and its components. Conversely, greater susceptibility was observed in the early stages, with 18 and 10 DEGs solely downregulated in the “cell wall” and “extracellular apoplast” regions (GO:0005576 and GO:0044421), respectively, as well as the “protein-containing complex” (GO:0032991). At the molecular function level, downregulated DEGs encoding catalytic activity (48 DEGs, GO:0003824), transcription regulators (11 DEGs, GO:0140110, GO:0003700), and “antioxidant activity” (29 DEGs, GO:0016209, GO:0004601). In contrast, a limited number of genes encoding transporters (3 DEGs, GO:0098772, GO:0048037) and regulatory functions (7 DEGs, GO:0098772, GO:0030234) were significantly upregulated (Figure 2a). Consistent with GO enrichment, KEGG analysis showed upregulation of more genes (48 DEGs) associated with defense, mainly encoded for “metabolic pathways” (cam01100), “biosynthesis of secondary metabolites” (cam01110), and “carotenoid biosynthesis” (cam00906). Moreover, downregulated DEGs were enriched in protein processing in the endoplasmic reticulum (18 DEGs, cam04141), plant hormone signal transduction (12 DEGs, cam04075), and flavonoid biosynthesis (5 DEGs, cam00941) (Figure 3a).

At 12 dpi, the pattern of the expression profile varied and showed a marked shift toward downregulation compared to 7 dpi, with only a few exhibiting significant upregulation. For instance, BP–GO enrichment revealed more upregulation of “cellular and metabolic processes,” suggesting an ongoing defense response. Furthermore, the complete downregulation of 38, 24, and 6 important genes associated with “response to stimulus” (GO:0050896), “regulation of biological processes” (GO:0050789), and “signaling” (GO:0023052 and GO:0007165) were observed at this stage. Additionally, under the condition S_7 vs S_12, 18 DEGs encoding “localization” (GO:0051179) were significantly underexpressed. Moreover, to overcome infection, essential genes were expressed more in the “membrane” (GO:0016020), whereas 20 DEGs associated with cell parts (GO:0005623 and GO:0044464) and extracellular regions (GO:0005576 and GO:0044421) were downregulated, further repressing the expression of 14 (11 + 3) genes associated with “protein synthesis” (GO:0032991). However, at the molecular function level, genes associated with the activities of “catalysis” and “binding” showed reduced expression under both C_12 vs S_12 and S_7 vs S_12 conditions compared to 7 dpi, indicating their significance in regulating susceptibility to infection. Perhaps, the key genes encoding “antioxidant activity” impacting the overexpression of transcription regulators (GO:0140110 and GO:0003700) could have declined the antioxidant activity (GO:0016209 and GO:0004601) during the prolonged stage of infection (Figure 2b). This is further lined with the susceptibility ratio between the 7 and 12 dpi (S_7 vs S_12) where, a total of 89 DEGs associated with “catalytic activity” in turn regulated by 23, 3 and 3 DEGs encoding for “transcription factors,” “transportation,” and “molecular function regulators” respectively overexpressed, indicating their signatures in downregulating 7 DEGs responsible for “antioxidant activities” (Figure 2c). Meanwhile, the KEGG pathway analysis further revealed downregulation at 12 dpi. A total of 18 DEGs (7 + 11) associated with “flavonoid biosynthesis” (cam00941) and “plant hormone signal transduction” (cam04075), showed higher fold enrichment than that observed at 7 dpi. Interestingly, at S_7 vs S_12, 66 genes encoding “biosynthesis of secondary metabolites” were completely downregulated, which implied the progression of infection from 7 to 12 dpi. Additionally, 14 pathways were downregulated, with a maximum DEGs (80) encoding metabolic (cam01100) and 9 DEGs encoding starch and sucrose metabolism (cam00500). In contrast, only one pathway associated with protein processing in the endoplasmic reticulum (cam04141) was upregulated, which was conferred by 16 DEGs (Figure 3b and c).

Across all three conditions (C_7 vs S_7, C_12 vs S_12, and S_7 vs S_12), no commonly upregulated DEGs were identified. In contrast, 9 DEGs (LOC101512924, LOC101504146, LOC101492217, LOC101489106, HSFB5, LOC101495272, LOC101489699, LOC113784091, and LOC113784643) were consistently downregulated across all conditions. Further, functional enrichment of these common DEGs revealed significant association with the ethylene-activated signaling pathway, molecular function regulator and DNA-binding transcription factor activity (Figure 1e–g; Supplementary File, S2).

### Expressed CAZymes, resistance genes, and transcription factors during Foc–chickpea interaction

#### Cell wall-related genes

**Figure 4.**
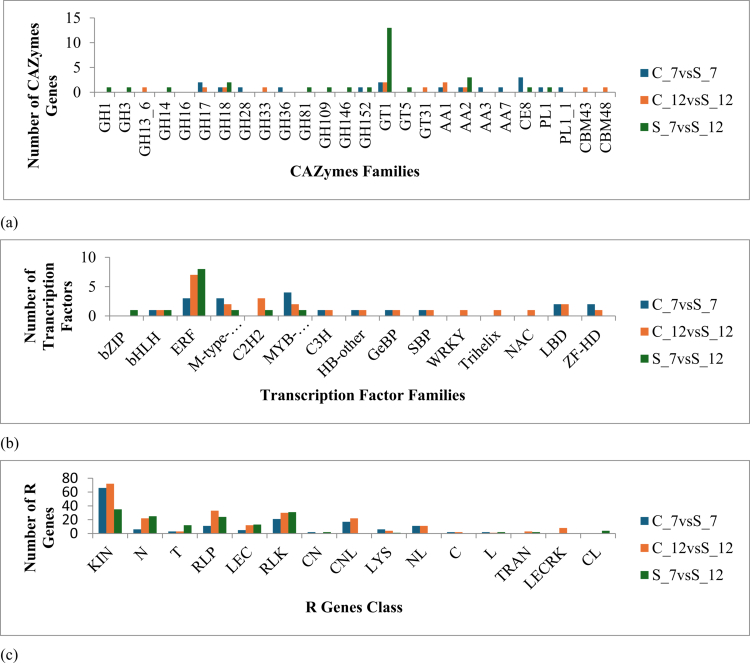
Comparative distribution of (a) CAZymes, (b) transcription factors, and (c) R genes identified from the DEGs. C_7 vs S_7: control vs stressed (Foc-infected) conditions on 7 d post inoculation; C_12 vs S_12: control vs stressed conditions on 12 d post inoculation and S_7 vs S_12: harvest on 7 d stress compared with 12 d stressed.

Polysaccharides such as cellulose, hemicellulose, and pectin constitute the main components of the plant cell wall and represent the primary barrier against pathogen attack.^26^ DEGs associated with cell wall proteins, particularly carbohydrate-active enzymes (CAZymes), were identified in Foc-infected chickpea across all comparisons. A total of 49, 43, and 47 CAZyme-encoding DEGs were detected in C_7 vs S_7, C_12 vs S_12, and S_7 vs S_12 conditions, respectively. These DEGs were classified into various modules, including auxiliary activities (AA), carbohydrate binding modules (CBM), carbohydrate esterases (CEs), glycoside hydrolase (GH), and glycosyl transferase (GT) (Supplementary File, S3). In C_7 vs S_7, CAZyme enrichment was restricted to specific families, with exclusive representation of auxiliary activities 3 and 7 (AA3, AA7, and AA7_e4) and glycoside hydrolase 28 (GH28) whereas, in C_12 vs S_12, exclusively enrichment of carbohydrate binding modules 43 and 48 (CBM43, CBM43_e49, and CBM 48), along with multiple glycoside hydrolase families, including GH13_6, GH13_e143, GH13_8, GH16_e232, and GH33), were identified. Moreover, the S_7 vs S_12 condition revealed a distinct CAZyme profile with exclusive expression of glycosyl transferase 5 (GT5 and GT5_e13), glycoside hydrolase 1, 3, 14, 81, and 146 (GH1, GH3, GH3_e0, GH14, GH14_e0, GH81, GH81_e4, and GH146) (Supplementary File, S7D; Figure 4a).

#### Transcription factor associated genes

At 7 dpi (C_7 vs S_7), a total of 19 DEGS encoding TFs were identified, associated with the bHLH, LBD, ZF-HD, GeBP, SBP, ERF, M-type_MADS, HB-other, C3H, and MYB-related families. Among these domains, the MYB-related domain was the most abundant, followed by the ERF and M-type_MADS TF. In contrast, bHLH, GeBP, and SBP were upregulated at this stage, while all other TFs were downregulated. Meanwhile, on C_12 vs S_12, 25 differentially expressed TFs belonged to LBD, ZF-HD, NAC, Trihelix, WRKY, SBP, GeBP, bHLH, ERF, M-type_MADS, HB-other, C3H, C2H2, and MYB-related with 4 TF domains, such as C2H2, WRKY, Trihelix, and NAC, were exclusively detected at 12 dpi. However, ERF constituted the largest TF family at this stage and was largely downregulated, whereas SBP domain remained upregulated. In contrast to those at 7 dpi, bHLH, and GeBP TFs were previously upregulated and downregulated at 12 dpi. Moreover, in S_7 vs S_12, 13 DEGs identified were associated with the bZIP, bHLH, ERF, M-type_MADS, C2H2, and MYB-related families. ERF domains were the most represented family in this comparison wherein, bZIP TFs were upregulated, while all other TFs families showed downregulated expression patterns (Figure 4b; Supplementary File, S4).

#### Resistance genes

152 and 223 DEGs from C_7 vs S_7 and C_12 vs S_12 conditions were identified as R-genes belonging to 12 and 13 different classes, respectively, such as KIN (kinase domain), N (only nucleotide binding site), T (only Toll/interleukin-1 receptor/TIR domain), RLP (receptor-like protein), LEC (lectin-like receptor), RLK (receptor-like kinase), TRAN (only a transmembrane helix domain), L (leucine-rich repeats), CNL (coiled-coil nucleotide-binding leucine-rich repeat), LYS (LysM-containing receptor), LEC (lectin receptor kinase), NL (nucleotide-binding leucine-rich repeat), and C (coiled-coil domain). This was based on various domains like TM (transmembrane), kinase, CC (coiled-coil), NBS (nucleotide binding site), LRR (leucine-rich repeat), LYSM (lysine motif), LECM (lectin motif), and TIR (Toll/interleukin-1 receptor), with two additional families of R-genes (CL (coiled-coil and LRR domains) and CN (coiled-coil and NBS domains) expressed exclusively under conditions C_7 vs S_7. In contrast, under the S_7 vs S_12 condition, 151 DEGs encoding R-genes belonged to 11 major classes, including KIN, N, T, RLP, LEC, RLK, CL, CN, L, LYS, and TRAN. Notably, the same number of domains were reported under the conditions C_7 vs S_7 and C_12 vs S_12 (Figure 4c).

### Protein–protein interaction and hub genes analysis

**Figure 5.**
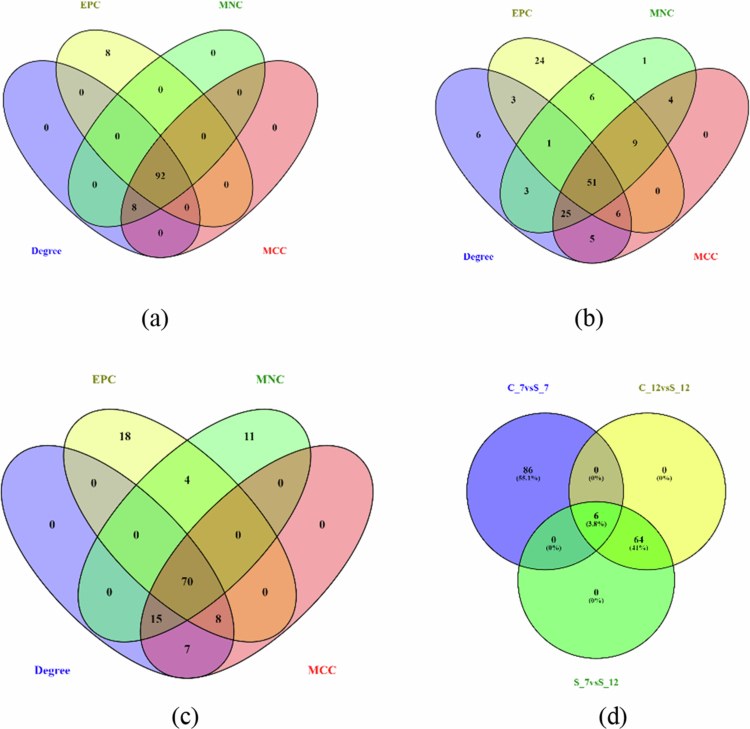
Interaction pattern of proteins represented for the corresponding top 100 hub genes (a) C_7 vs S_7; (b) C_12 vs S_12; (c) S_7vsS_12; and (d) overall commonly shared genes among different time points. C_7 vs S_7: control vs stressed (Foc-infected) conditions on 7 d post inoculation; C_12 vs S_12: control vs stressed conditions on 12 d post inoculation and S_7 vs S_12: harvest on 7 d stress compared with 12 d stressed. The comparison of the interaction pattern for the 100 top-ranked hub genes revealed that a maximum number of them formed a network on the 7 dpi which declined on the 12 dpi due to Foc infection. EPC (yellow): edge percolated component; MNC (green): maximum neighborhood component; and MCC (red): maximal clique centrality.

**Figure 6.**
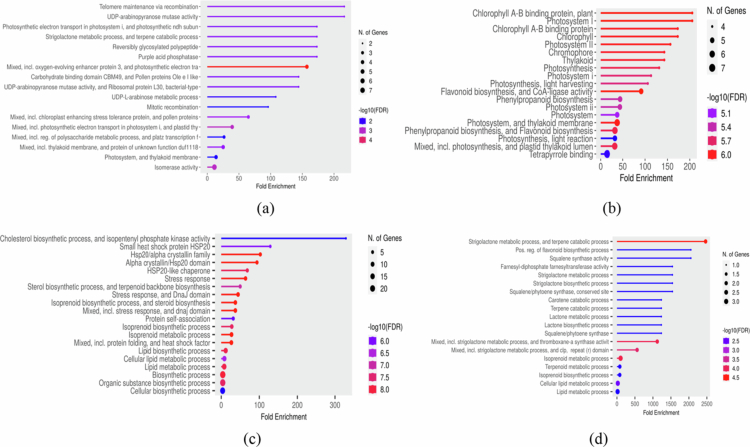
The functional enrichment during the interaction is represented through ShinyGO (a) C_7 vs S_7; (b) C_12 vs S_12; (c) S_7 vs S_12; and (d) common hub genes among different time points. C_7 vs S_7: control vs stressed (Foc-infected) conditions on 7 d post inoculation; C_12 vs S_12: control vs stressed conditions on 12 d post inoculation and S_7 vs S_12: harvest on 7 d stress compared with 12 d stressed.

The topological properties of the network under C_7 vs S_7 comprised a total of 110 nodes and edges (Table 1). Additionally, sub-clustering of the network identified nine distinct clusters, of which the two highest scoring clusters were selected for further analysis. Cluster 1 exhibited a score of 4.50 and contained five nodes connected by nine edges, including “uncharacterized LOC101501852,” “peroxidase 7-like” (LOC101512456 and LOC101514192), and “40S ribosomal protein” (LOC101497545 and LOC101504926). Besides, cluster 2, showed a score of 4.00 and consisted of four nodes and six edges representing genes encoding photosynthetic NDH subunits (LOC101515473, LOC101504033, LOC101497210, and LOC101494683). Notably, all the genes within both clusters were upregulated (Supplementary File, S5 and S6A). Hub gene analysis of the PPI network identified 92 common genes with functional enrichment, indicating that most genes (seven) were involved in “isomerase activity” and in the “photosystem,” and “thylakoid membrane” (five genes) (Figures 5a and 6a, Supplementary File, S7A). Additionally, mapping of DEGs against the UniProt database revealed only one upregulated protein (UniProt ID: 49816) under C_7 vs S_7 conditions, whereas no upregulated proteins were identified under C_12 vs S_12. In contrast, eight downregulated proteins were identified of which seven proteins were common between the two conditions (UniProt IDs: B5LMN4, B5LMM8, B5LMS3, B5LMR9, B5LMS0, B5LMS9, and B5LMS4). An additional protein, Q5YK05, was uniquely downregulated at 7 d while B5LMN4 was exclusive to 12 dpi. No proteins were observed under the S_7 vs S_12 condition (Supplementary File, S8).

**Table 1.** Topological properties of the connected network.

Description	C_7 vs S_7	C_12 vs S_12	S_7 vs S_12
Number of nodes	110	276	195
Number of edges	110	462	337
Average number of neighbors	2.875	3.854	4.569
Network diameter	6	22	13
Network radius	3	11	7
Clustering coefficient	0.283	0.266	0.391
Connected components	27	23	23

C_7 vs S_7: control vs stressed (Foc-infected) conditions on 7 d post inoculation; C_12 vs S_12: control vs stressed conditions on 12 d post inoculation and S_7 vs S_12: harvest on 7 d stress compared with 12 d stressed.

Under the C_12 vs S_12 condition, the PPI network comprised of 276 nodes connected by 462 edges, as revealed by topological analysis (Table 1). The sub-clustering of the network identified 17 distinct clusters, of which the two highest scoring were selected for further analysis. Cluster 1 achieved a score of 4.50 and consisted of five nodes and nine edges and included two upregulated uncharacterized genes, “LOC101494181” and “LOC105851647,” along with three downregulated genes encoding “putative ethylene insensitive 3-like” four proteins (LOC101500668), “LOB domain-containing protein 25-like isoform X2” (LOC101490681) and the protein “SAR deficient 1-like” (LOC101500982). However, cluster 2, exhibited a score of 4.00 with four nodes connected by six edges, consisting of “Casparian strip membrane protein 1-like” (LOC101512299), which was upregulated, and three downregulated genes encoding “heavy metal-associated isoprenylated plant protein 7-like isoform X2” (LOC101507929), “non-specific lipid-transfer protein 1-like” (LOC101495082), and “heavy metal-associated isoprenylated plant protein 12-like” (LOC113784545) (Supplementary File, S5 and S6B). Additionally, hub gene analysis identified 51 common genes across the connectivity of degree, MCC, MNC, and EPC. The functional enrichment of these hub genes showed significant representation of “tetrapyrolle binding,” “flavonoid biosynthesis,” “CoA-ligase activity,” “phenylpropanoid biosynthesis,” and “photosynthesis” processes (Figures 5b and 6b; Supplementary File, S7B).

The topological properties in the network of S_7 vs S_12 revealed 195 nodes and 337 edges (Table 1), with 13 distinct clusters. Cluster 1 consisted of eight nodes and 27 edges, yielding a score of 7.714. These genes included “probable oxidosqualene cyclase” (LOC101513129), “beta-amyrin synthase” (LOC101507549), “farnesyl pyrophosphate synthase 1” (LOC101497550), “hydroxymethylglutaryl-CoA synthase” (LOC101507987), “isopentenyl-diphosphate delta-isomerase I” (LOC101509488), “diphosphomevalonate decarboxylase MVD2, peroxisomal” (LOC101491864), “heterodimeric geranylgeranyl pyrophosphate synthase large subunit 1, chloroplastic” (LOC101490130), and “phytoene synthase 2” (LOC101509318), of which only LOC101513129 was upregulated. Cluster 2 showed a score of 5.600 and comprised six nodes with 14 edges and included “NAC transcription factor protein 2” (NAC2), which was the only upregulated gene, along with several downregulated genes, including “allene oxide cyclase,” “chloroplastic-like” (LOC101504350 and LOC101500382), protein “TIFY 10a-like” (LOC101491099), “probable glycerol-3 phosphate acyltransferase 2” (LOC101498386), and “abscisic acid receptor P4L4-like” (LOC101509736) (Supplementary Files S5 and S6C). Moreover, hub gene analysis identified 70 common genes amid the four analysis parameters. The functional enrichment of these hub genes indicated significant association with “cellular biosynthetic process,” “cellular lipid metabolic process,” “stress response,” and “isoprenoid biosynthetic” processes (Figures 5c and 6c; Supplementary File, S7C).

Interestingly, common of common hub genes across 7 dpi, 12 dpi, and S_7 vs S_12 revealed a total of 6 hub genes associated with “nitrate reductase [NADH]-like” (LOC101498580), “phytoene synthase 2, chloroplastic-like,” “transcript variant X1” (LOC101509318), “heterodimeric geranylgeranyl pyrophosphate synthase large subunit 1, chloroplastic” (LOC101490130), “carotenoid cleavage dioxygenase 8 homolog B, chloroplastic isoform X1” (LOC101497957), “beta-carotene isomerase D27, chloroplastic-like” (LOC101489176), and “shikimate O-hydroxycinnamoyltransferase-like” (LOC101502383). The functional enrichment identified various processes like “terpenoid metabolic,” “lipid metabolic,” and “flavonoid biosynthetic” processes, as also represented in Figures 5d and 6d (Supplementary File, S7D).

## Discussion

Over the years, transcriptome studies have been widely applied to detect infections and decipher the early mechanisms of plant‒pathogen interactions by exploring transcription factors and dynamic gene expression patterns during plant development.^5^^,^^8^ In the present investigation, DEG analysis revealed complex transcriptional reprogramming in response to *F. oxysporum* f. sp. *ciceris* (Foc) infection, with several genes associated with cellular, molecular, and biological processes and metabolic pathways that are differentially regulated at two infection stages (7 and 12 dpi). GO analysis at both time points revealed an escalation in the expression of regulated genes as the disease progressed, reflecting a dynamic host response to prolonged pathogen stress, as illustrated in Figure 2. Perhaps, a comparative analysis of the S_7 vs S_12 condition revealed aberrant expression, particularly the progressive decline of certain genes whose reduced expression adversely impacted multiple biological and cellular functions, including immune responses, as similarly reported in tomato and banana.^27^^,^^28^ Importantly, the consistent downregulation of nine key genes across infection stages, which were associated with plant growth, development, and stress-responsive signaling pathways, suggested a compromised defense potential under prolonged *F. oxysporum* infection.^29^

Furthermore, the GO and KEGG enrichment analyses indicated a pronounced upregulation of the pectin catabolism process, inferring degradation of the homogalacturonan subunit of pectin in the host plant cell wall during early infection (7 dpi), thus facilitating fungal entry, which was consistent with prior findings.^26^ Concurrently, the host appeared to activate compensatory mechanisms, as evidenced by the upregulation of genes encoding peroxidases, the DNA repair protein RAD50, abscisic acid 8′-hydroxylase 3-like, and beta-amyrin 11-oxidase-like enzymes. These genes, implicated in cell wall modification, biogenesis, and sterol metabolism, were upregulated, implying a mechanism to sustain plant cell wall integrity, with plant sterols regulating seedlings' root growth and development. This finding aligns with earlier studies in *Arabidopsis thaliana* and *Populus deltoides*, where galacturonosyltransferase (GAUT) proteins (AtGAUT1 and AtGAUT7) have been shown to mediate homogalacturonan (HG) synthesis, which is required for primary cell wall formation in actively growing root tissues, thereby influencing the gene effect.^30^^,^^31^ Since the plant cell wall constitutes a primary physical barrier against fungal invasion, its reinforcement remains a critical defense, especially as fungi secrete a wide range of CWDEs that can trigger host defense gene expression.^26^^,^^32^

Analysis of CAZymes further substantiated the destructive Foc strategy during the early stages of infection. At 7 dpi, strong expression of the GH family was observed, followed by the AA family, signifying their role in degrading α-D-galacturonic acid residues, a major component of pectin as well as cellulose. These enzymatic activities generate oligosaccharides that not only serve as nutritional substrates for the fungus but also function as elicitors of host defense responses, reflecting a dual role in pathogenicity and immune signaling.^31^^,^^33^^,^^34^ As the infection advanced, fungi appeared to adopt unobtrusive pathogenic strategies to evade detection by the host immune system, as suggested by the enhanced release of alpha‒amylase at 12 dpi (C_12 vs S_12), a phenomenon previously associated with a stealthy colonization mechanism.^35^ Additionally, comparative analysis of S_7 vs S_12 determined increased expression of cell-wall hydrolases such as xylosidase, which degrades hemicellulose into oligocarbohydrates that serve as substrates for virulence-associated enzymes, as also reported for the fungi *Verticillium dahliae*^36^ and *F. oxysporum.*^37^ Similarly, other enzymes, including glucanase, xylanase, and chitinase, previously reported in chickpea^12^ and pea,^34^ were also identified, reinforcing their established roles in plant‒pathogen interactions.

Furthermore, the analysis highlighted a significant role of Cajal bodies (CB), with genes associated with CB formation (GO:0015030) showing marked upregulation during infection. This finding substantiates the regulatory involvement of CBs in coordinating cell wall synthesis, RNA formation, maturation, assembly and DNA repair processes even under severe biotic stress, thereby supporting plant growth and development during pathogen challenge.^38^^,^^39^ Additionally, genes associated with the plant cell wall (GO:0009505), membrane (GO:0016020), and chloroplast formation (GO:0009507) were upregulated, implying their collective involvement in conferring FW resistance. This inference was further evidenced by the enhanced expression of MFs such as pectinesterase inhibitor activity and magnesium ion transmembrane transporter activity, both of which contribute to cell wall stabilization and the biosynthesis of defense-related substances,^26^ in accordance to the observations supported by earlier research in banana,^25^ mung bean^40^ and horticulture plants.^41^ Overall, these responses reflect a coordinated involvement of BP, MF, and CC in establishing early defense mechanisms against Foc infection.

As infection progressed to 12 dpi, transcriptomic changes extended beyond cell wall and nuclear functions, encompassing genes linked to photosynthesis, cell division and defense pathways, suggesting sustained efforts by the host to maintain growth despite escalating infection. Photosynthesis is increasingly recognized as a critical contributor to plant resistance and stress management, even during fungal infection invasion, as previously evidenced in wheat plants.^42^ Concomitantly, enhanced expression of genes (GO:0016117) involved in carotenoid biosynthesis was observed, influencing plastid formation (GO:0009536) and providing photoprotection to the light-harvesting system (photosynthesis) under reactive oxygen species (ROS) stress.^43^ This was accompanied by upregulation of genes associated with protein phosphorylation (GO:0006468), a key post-translational modification (PTM) that modulates immune responses by phosphorylating serine, threonine and tyrosine residues, regulating the MAPK signaling cascades, consistent with observations in *Arabidopsis.*^44^ Moreover, genes involved in the mannose metabolism (GO:0006013) were upregulated during early infection (7 dpi), corresponding with fungal secretion of mannitol to quench the ROS and modulate host defenses, similar to *Alternaria alternata* infection in tobacco.^45^ The increased expression of MFs, such as farnesyltransferase and geranylgeranyl-diphosphate geranylgeranyltransferase activities, further emphasized their roles in regulating plant growth and pathogen defense.^46^ The upregulated ABC-type transporters also supported immune responses by facilitating the transport of phytohormones and heavy metals, consistent with findings in wheat crop infected by *Fusarium graminearum.*^47^

Overall, these observations suggest that early photosynthetic responses may divulge plants' capacity to withstand biotic stress, as reflected in the S_7 vs S_12 condition, where upregulated genes (GO:0032501, GO:0005488, and GO:0009987) indirectly influenced photosynthesis and carotenoid production (secondary metabolites), thereby sustaining seedling growth and development, as previously evidenced in *Linum usitatissimum.*^48^ This was further supported by the overexpression of homeostasis-related genes (GO:0051787, GO:0016887, GO:0031072, and GO:0140662) associated with misfolded protein binding, ATP hydrolysis, heat shock protein (HSP) binding and ATP-dependent protein folding chaperone activity. These genes are indicative of an unfolded protein response (UPR) triggered by the accumulation of misfolded proteins in the endoplasmic reticulum during disease progression from 7 to 12 dpi, which is consistent with the findings of similar studies.^49^^,^^50^ Meanwhile, enhanced expression of antioxidant-related genes, including those involved in hydrogen peroxide (H_2_O_2_) catabolism, further suggested active mitigation of oxidative stress induced by fungal hyphal spread.^51^ Despite these adaptive responses, prolonged Foc infection likely compromises the plant immune system.^52^

Resistance (R) genes plays a pivotal role in pathogen detection and defense activation, which are typically enriched with NBS and LRR domains.^53^ In the present study, eight R-gene domains—TM, kinase, NBS, TIR, LRR, LECM, CC and LYSM were identified from early infection stages onward, with an additional CN class expressed exclusively at 7 dpi, contributing to pathogen recognition and systemic resistance activation.^53^ Similar R-genes have been reported in strawberries infected with *F. oxysporum* f. sp. *fragariae*, indicating a conserved resistance mechanism across diverse plant species.^54^

While several defense-associated genes were upregulated, a substantial subset showed significant downregulation at 7 and 12 dpi relative to their respective controls. These included BPs such as protein complex oligomerization, ethylene-activated signaling pathways, systemic acquired resistance, and auxin response, all of which declined progressively, indicating a weakening immune state.^29^ Downregulation of flavonoid, jasmonic- and isoprenoid-biosynthetic processes at S_7 vs S_12 further suggested weakened immune capacity, highlighting reduced phytohormones levels and secondary metabolite production essential for defense, in line with earlier studies in chickpea^55^ and flax.^48^ Additionally, downregulation of proton transmembrane transporter and acyltransferases involved in nutrient allocation, membrane synthesis, lipid storage and ion homeostasis suggested impaired cellular signaling and metabolic balance.^56^^,^^57^ Perhaps, reductions in transporter activity may hinder nutrient immobilization, as previously reported in barley, where inhibition of phytase enzymes of *Fusarium* and *Aspergillus* by aspartic-type endopeptidase enhanced host defense responses.^58^

The most pronounced downregulation in S_7 vs S_12 occurred among MFs such as terpene synthase, dioxygenase, and O-methyltransferase activities, signifying reduced phytohormone biosynthesis.^48^ KEGG pathway analysis further confirmed suppression of flavonoid biosynthesis and plant hormone signal transduction pathways, indicating metabolic repression and impaired growth under advancing fungal infection.^55^ Several transcription factors (TFs) such as MYB, homeodomain leucine zipper, zinc finger protein, and helix–loop–helix were identified, corroborating earlier reports in chickpea–Foc interaction.^10^ Differential regulation of TF families such as C3H, ERF, SBP, GeBP, and LBD predominantly underexpressed at 12 dpi suggested compromised phytohormone synthesis. Additionally, the reduced expression of TFs such as C2H2, WRKY, trihelix, and NAC at 12 dpi likely contributed to disease progression through disrupted ROS homeostasis and reduced root resilience.^59^ A key finding of our study is the downregulation of multiple bHLH and GeBP TFs at 12 dpi. This finding is highly significant, as these genes are central regulators of flavonoid biosynthesis and cytokinin-mediated defense signaling, respectively. Rather than being a simple marker of disease progression, these findings could represent an active strategy by Foc to suppress a critical branch of the chickpea immune system, a phenomenon observed in other pathosystems.^60^^,^^61^ These results pinpoint the bHLH pathway as a crucial battleground during late-stage interaction between chickpea and Foc. Moreover, genes involved in starch and sucrose metabolism were downregulated in S_7 vs S_12, indicating diminished carbohydrate availability for defense, a finding similar to those in tomatoes infected by *F. oxysporum*. Interestingly, specific upregulation of bZIP TFs in S_7 vs S_12, likely supporting hormone-mediated signaling pathways, including abscisic acid, jasmonic acid, ethylene, and salicylic acid pathways, as well as oxidative defense responses, as previously reported in soybean to aid resistance against *Sclerotinia sclerotiorum* and *Phytophthora sojae.*^62^

Network analysis further demonstrated a temporal shift in transcriptional priorities, with DEGs encoding cellular transporters and regulatory enzymes predominating at 7 dpi, while those encoding TFs and lipid-binding elements were more prominent at 12 dpi. Under the S_7 vs S_12 condition, the DEGs responsible for secondary metabolite biosynthesis and signaling pathways formed an interconnected defense network, with sugars serving as internal signal modulators.^10^ Our findings suggested that increasing infection utilizes the plant's potential for TFs, influencing R-genes to regulate common target pathways.^63^

PPI network analysis revealed a significant shift in metabolic priorities between the two time points. At 7 dpi, the hub genes were strongly enriched in photosystem and thylakoid membrane-associated processes, and the downregulation of many of these genes suggested an early suppression of photosynthesis in response to infection. By 12 dpi, the network focus shifted markedly toward secondary metabolism, with significant enrichment of flavonoid and phenylpropanoid biosynthesis pathways, indicating a critical reallocation of resources from growth towards the production of defense compounds. Six hub genes common across all conditions (C_7 vs S_7, C_12 vs S_12, and S_7 vs S_12) were associated with terpenoid biosynthesis, lipid metabolism, and ABA synthesis, indicating their roles in activating early defense responses to combat Fusarium infection.^48^ These hub genes represent promising candidates for further functional characterization aimed at improving wilt resistance in chickpea and other leguminous crops.

Moreover, DEGs mapped against UniProt revealed a notable expression pattern of “Late embryogenesis abundant protein 1” (CapLEA-1), which was upregulated at 7 dpi, contributing to water deficit tolerance.^64^ “Maturase K,” a group II intron, was exclusively downregulated at 7 dpi, indicating potential disruptions in gene expression regulation.^65^ At 12 dpi, selective downregulation of “Small ribosomal subunit protein uS14c,” essential for protein synthesis during plant development, was observed, consistent with findings in rice and maize.^30^ Notably, these predicted proteins did not overlap with MCODE sub-clusters or hub genes, suggesting inadequate information about proteins involved in fungal defense and highlighting the need for further discovery and functional validation in this domain.

## Conclusion

The study revealed significant transcriptomic reprogramming and early defense activation in chickpea under Foc stress at 7 and 12 dpi, highlighting key metabolites, phytohormones, CAZymes, TFs, R-genes, and hub genes involved in regulating molecular mechanics during Foc–chickpea interaction. Commonly enriched DEGs across both time points were linked to cell-wall reinforcement, terpenoid synthesis and H₂O₂ catabolism, enhanced secondary metabolite and lipid biosynthesis and increased light-harvesting and magnesium transport activities. CAZyme profiling revealed the early secretion of GH28 and GH17 by Foc, indicating initial pathogenic activity, followed by increased glycosyl hydrolase expression with infection progression. The upregulation of the bZIP, GeBP, and TF families at 12 dpi suggested that the host's potential to combat disease progression providing late-stage tolerance with R-gene classes like KIN, RLP and RLK highly expressed since the onset of infection. PPI network analysis further emphasized the contribution of secondary metabolites and hormone-related DEGs to sustaining defense responses. Overall, this work provides clear insights into the molecular mechanisms underlying chickpea tolerance and adaptation during Fusarium wilt progression and identifies key hub genes as strong candidates for future functional validation and the development of markers to support wilt-resistance chickpea breeding programs.

## Supplementary Material

Supplementary material Supplementary_File_S6_MCODE.xlsx

Supplementary material Supplemetary_File_S5_Figures.docx

Supplementary material Supplementary_File_S4_TF.xlsx

Supplementary material Supplementary_File_S2_Common_DEGs.xlsx

Supplementary material Supplemetary_File_S1.docx

Supplementary material Supplementary_File_S8_ProteinID_Mapping.xlsx

Supplementary material Supplementary_File_S3_CAZymes.xlsx

Supplementary material Supplementary_File_S7_Hubgenes.xlsx

## Data Availability

Publicly available datasets were analyzed in this study and the accession ID is provided in the manuscript/supplementary material.
