# Supplementary material for: Computational studies deciphered the role of key genes and associated networks regulating the defense mechanism in chickpea under Fusarium oxysporum f. sp. ciceris induced wilt condition
Source: Plant Signal Behav. 2026 Feb 18;21(1):2631915. doi: 10.1080/15592324.2026.2631915 (PMC12928610; doi:10.1080/15592324.2026.2631915)
Supplement: Supplementary material — Supplemetary_File_S5_Figures.docx [file KPSB_A_2631915_SM7585.docx]

(a)
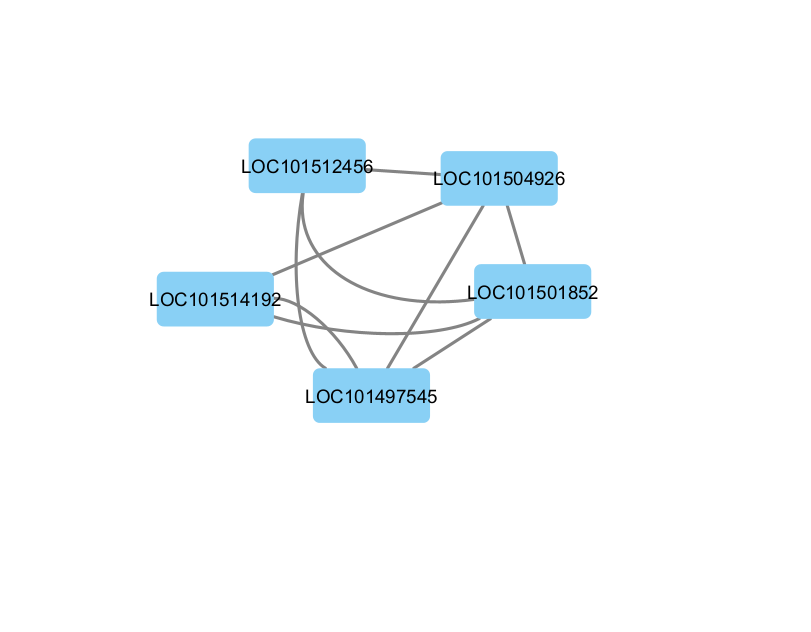

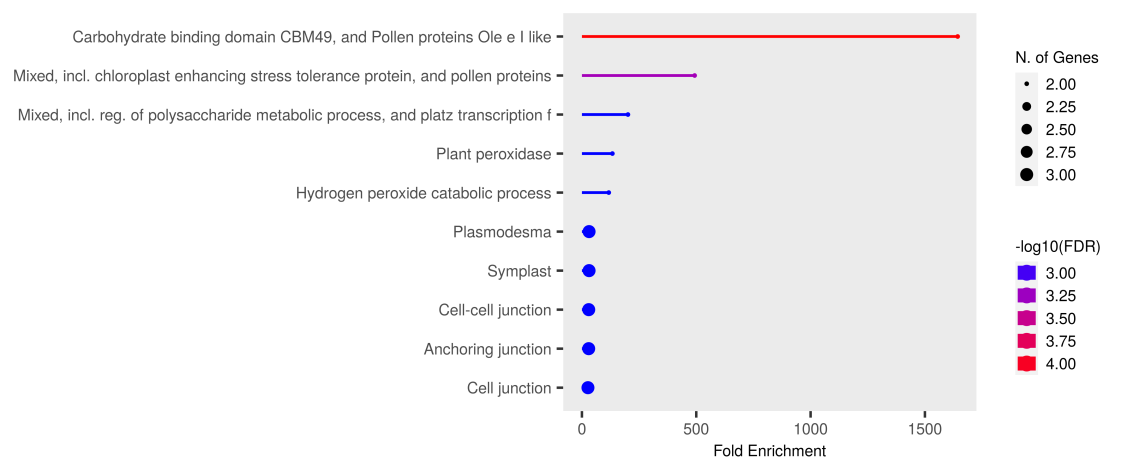
 (b)


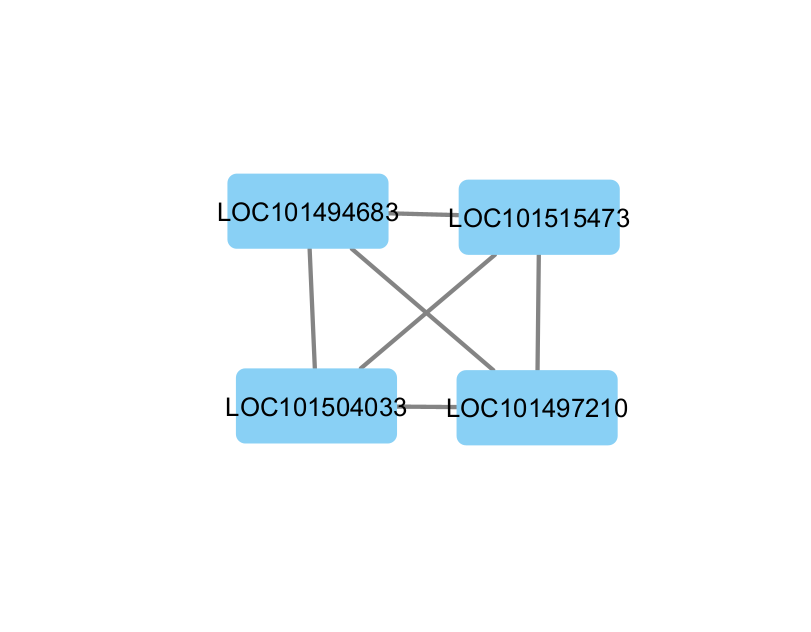

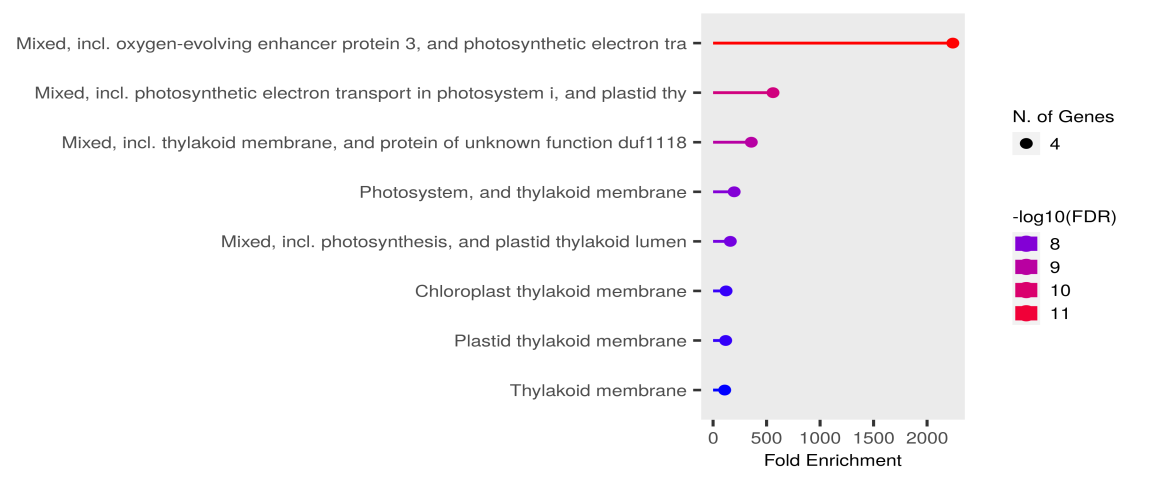


(c) (d)

Figure S1: Top 2 Sub-clustering MCODE networks and their biological process enrichment analysis in chickpea at 7dpi to Foc infection. (a) MCODE sub-cluster 1, (b) The biological process enrichment of sub-cluster 1, (c) MCODE sub-cluster 2 and (d) The biological process enrichment of sub-cluster 2.

1.
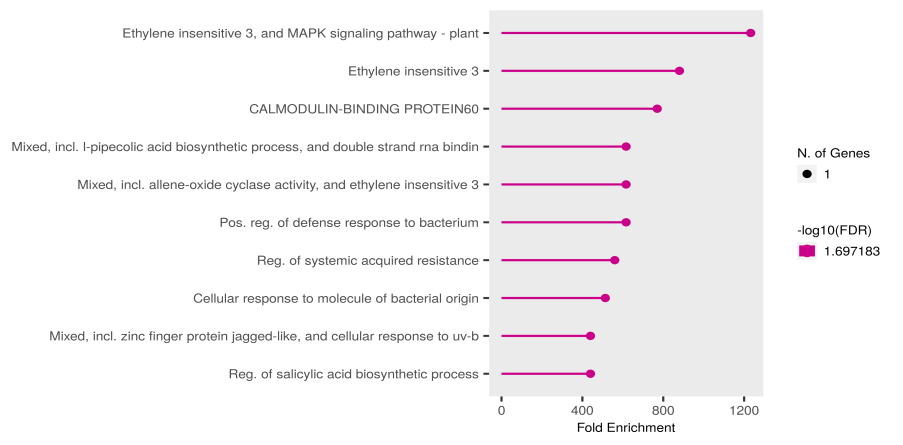

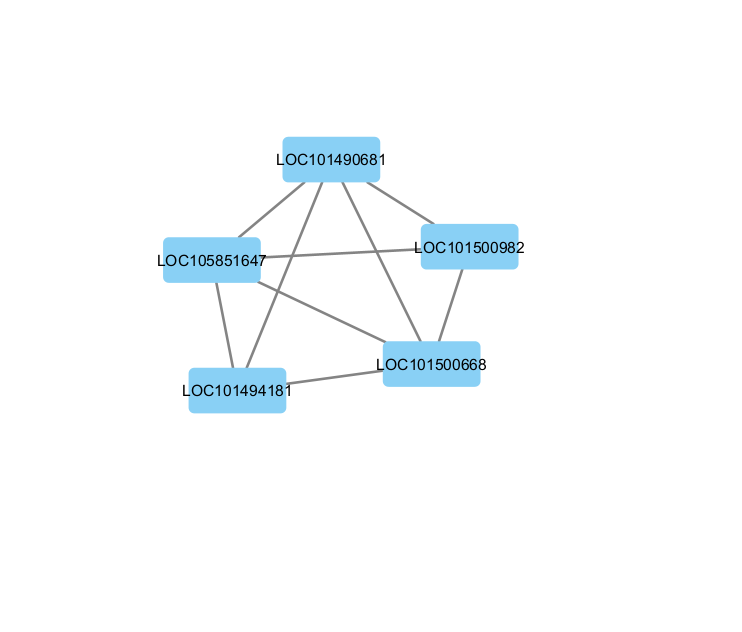
(b)


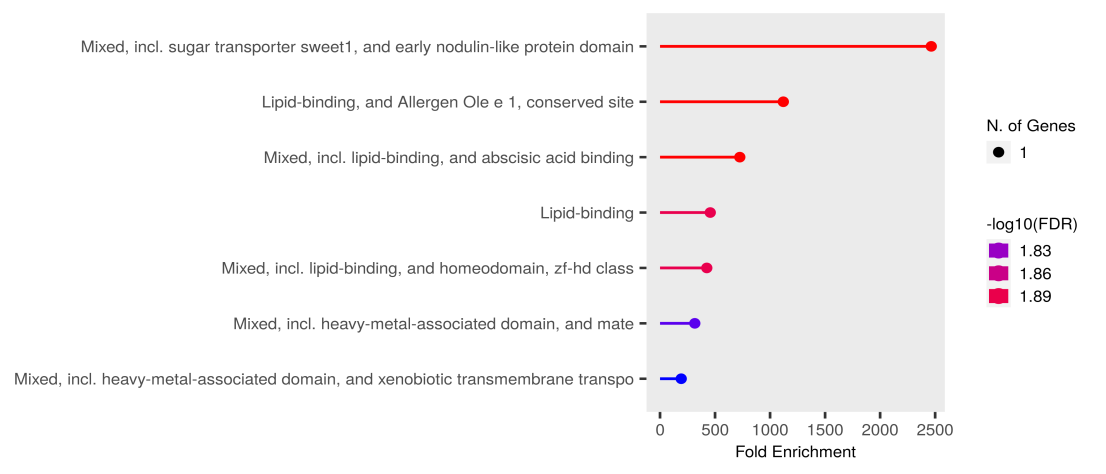

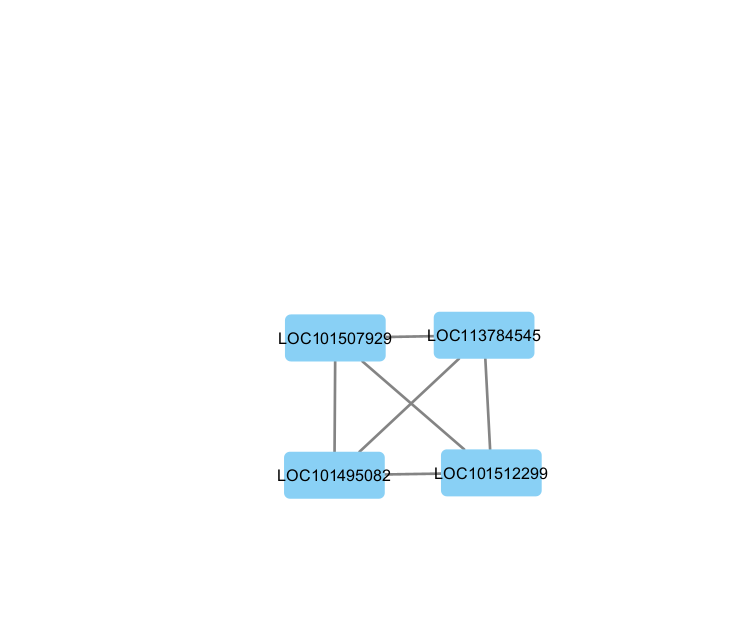


(c) (d)

Figure S2: Top 2 Sub-clustering MCODE networks and their biological process enrichment analysis in chickpea at 12dpi to Foc infection. (a) MCODE sub-cluster 1, (b) The biological process enrichment of sub-cluster 1, (c) MCODE sub-cluster 2 and (d) The biological process enrichment of sub-cluster 2.


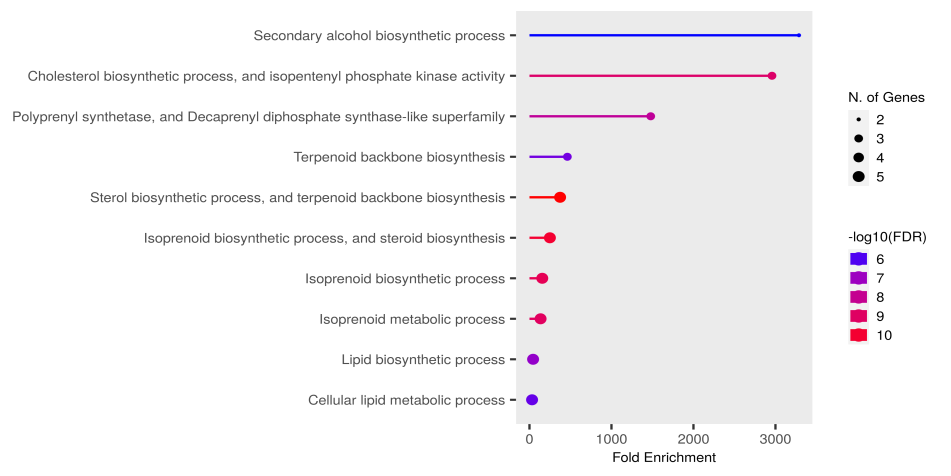

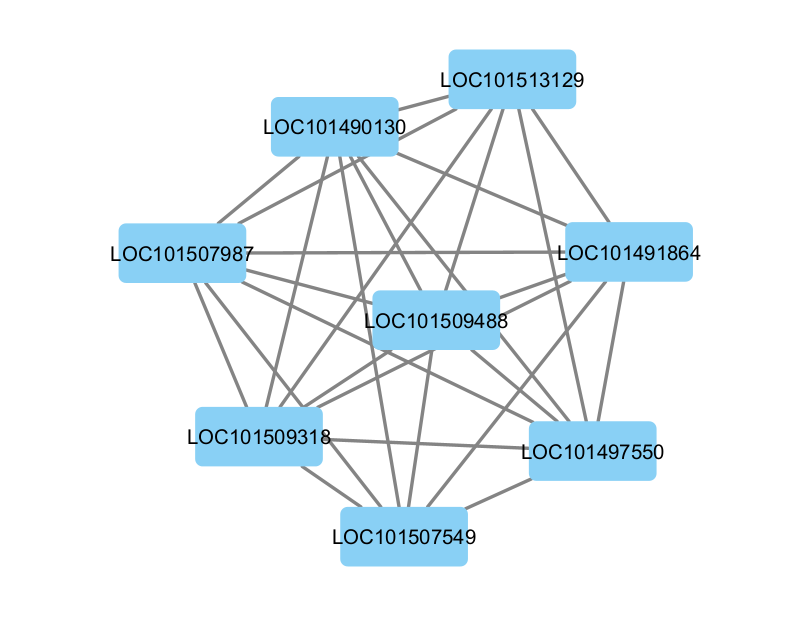


(a) (b)


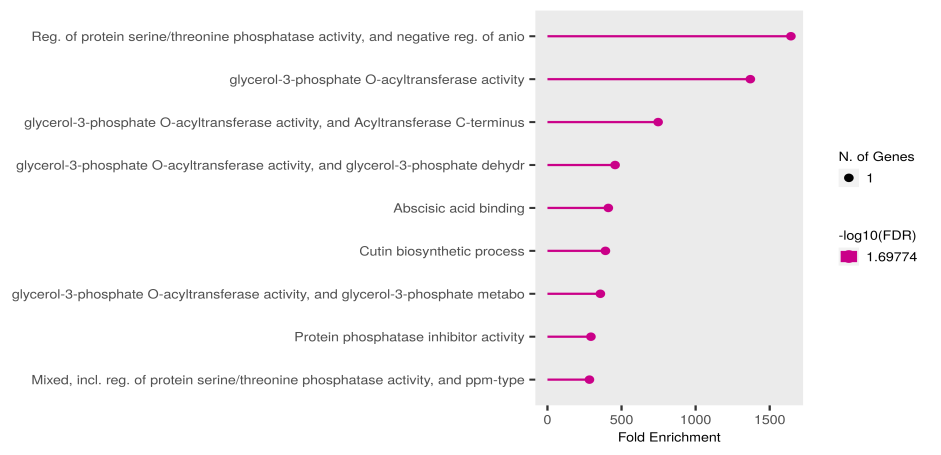

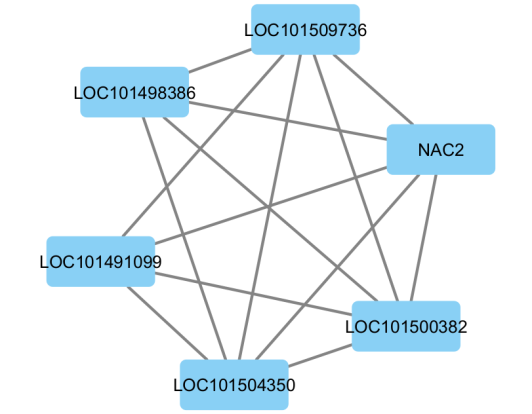


(c) (d)

Figure S3: Top 2 Sub-clustering MCODE networks and their biological process enrichment analysis in chickpea at S_7vsS)12. (a) MCODE sub-cluster 1, (b) The biological process enrichment of sub-cluster 1, (c) MCODE sub-cluster 2 and (d) The biological process enrichment of sub-cluster 2.
