# Supplementary material for: Computational studies deciphered the role of key genes and associated networks regulating the defense mechanism in chickpea under Fusarium oxysporum f. sp. ciceris induced wilt condition
Source: Plant Signal Behav. 2026 Feb 18;21(1):2631915. doi: 10.1080/15592324.2026.2631915 (PMC12928610; doi:10.1080/15592324.2026.2631915)
Supplement: Supplementary material — Supplemetary_File_S1.docx [file KPSB_A_2631915_SM7588.docx]

S1-Table 1: Experimental design with the dataset included for Differential Expression Gene (DEG) analysis

| **C_7vsS_7** | | **C_12vsS_12** | | **S_7vsS_12** | |
| --- | --- | --- | --- | --- | --- |
| WR315_C_7 | WR315_S_7 | WR315_C_12 | WR315_S_12 | WR315_S_7 | WR315_S_12 |
| ICCV05528_C_7 | ICCV05528_S_7 | ICCV05528_C_12 | ICCV05528_S_12 | ICCV05528_S_7 | ICCV05528_S_12 |
| C214_C_7 | C214_S_7 | C214_C_12 | C214_S_12 | C214_S_7 | C214_S_12 |
| JG62_C_7 | JG62_S_7 | JG62_C_12 | JG62_S_12 | JG62_S_7 | JG62_S_12 |
| 10057_C_7 | 10057_S_7 | 10057_C_12 | 10057_S_12 | 10057_S_7 | 10057_S_12 |
| 10058_C_7 | 10058_S_7 | 10058_C_12 | 10058_S_12 | 10058_S_7 | 10058_S_12 |

C_7vsS_7: control (C) versus stressed (S) at seventh dpi.

C_12vsS_12: control (C) stressed (S) at twelfth dpi

S_7vsS_12: stressed (S) at seventh versus stressed (S) at twelfth dpi

dpi: days post inoculation

S1-Table 2: Data information with alignment score

| **SAMPLE** | **CONDITION** | **TIME** | **ALIGNMENT SCORE (%)** |
| --- | --- | --- | --- |
| WR315_S_7 | *Fusarium oxysporum* f. sp. *ciceris* infected | 7dpi | 90.02 |
| WR315_C_7 | Control | 7dpi | 96.61 |
| WR315_S_12 | *Fusarium oxysporum* f. sp. *ciceris* infected | 12dpi | 92.31 |
| WR315_C_12 | Control | 12dpi | 95.38 |
| ICCV05528_S_7 | *Fusarium oxysporum* f. sp. *ciceris* infected | 7dpi | 91.85 |
| ICCV05528_C_7 | Control | 7dpi | 95.06 |
| ICCV05528_S_12 | Fusarium oxysporum f. sp. ciceris infected | 12dpi | 91.99 |
| ICCV05528_C_12 | Control | 12dpi | 97.11 |
| C214_S_7 | *Fusarium oxysporum* f. sp. *ciceris* infected | 7dpi | 93.78 |
| C214_C_7 | Control | 7dpi | 96.73 |
| C214_S_12 | *Fusarium oxysporum* f. sp. *ciceris* infected | 12dpi | 93.38 |
| C214_C_12 | Control | 12dpi | 96.96 |
| JG62_S_7 | *Fusarium oxysporum* f. sp. *ciceris* infected | 7dpi | 89.95 |
| JG62_C_7 | Control | 7dpi | 96.35 |
| JG62_S_12 | *Fusarium oxysporum* f. sp. *ciceris* infected | 12dpi | 93.43 |
| JG62_C_12 | Control | 12dpi | 96.12 |
| 10057_S_7 | *Fusarium oxysporum* f. sp. *ciceris* infected | 7dpi | 94.13 |
| 10057_C_7 | Control | 7dpi | 95.45 |
| 10057_S_12 | *Fusarium oxysporum* f. sp. *ciceris* infected | 12dpi | 92.32 |
| 10057_C_12 | Control | 12dpi | 95.96 |
| 10058_S_7 | *Fusarium oxysporum* f. sp. *ciceris* infected | 7dpi | 89.23 |
| 10058_C_7 | Control | 7dpi | 94.06 |
| 10058_S_12 | *Fusarium oxysporum* f. sp. *ciceris* infected | 12dpi | 93.07 |
| 10058_C_12 | Control | 12dpi | 96.70 |
